# Combined effects of glycan chain length and linkage type on the immunogenicity of glycoconjugate vaccines

**DOI:** 10.1038/s41541-021-00409-1

**Published:** 2021-12-10

**Authors:** Chakkumkal Anish, Michel Beurret, Jan Poolman

**Affiliations:** Bacterial Vaccines Discovery and Early Development, Janssen Vaccines and Prevention B.V., Leiden, Netherlands

**Keywords:** Immunology, Conjugate vaccines

## Abstract

The development and use of antibacterial glycoconjugate vaccines have significantly reduced the occurrence of potentially fatal childhood and adult diseases such as bacteremia, bacterial meningitis, and pneumonia. In these vaccines, the covalent linkage of bacterial glycans to carrier proteins augments the immunogenicity of saccharide antigens by triggering T cell-dependent B cell responses, leading to high-affinity antibodies and durable protection. Licensed glycoconjugate vaccines either contain long-chain bacterial polysaccharides, medium-sized oligosaccharides, or short synthetic glycans. Here, we discuss factors that affect the glycan chain length in vaccines and review the available literature discussing the impact of glycan chain length on vaccine efficacy. Furthermore, we evaluate the available clinical data on licensed glycoconjugate vaccine preparations with varying chain lengths against two bacterial pathogens, *Haemophilus influenzae* type b and *Neisseria meningitidis* group C, regarding a possible correlation of glycan chain length with their efficacy. We find that long-chain glycans cross-linked to carrier proteins and medium-sized oligosaccharides end-linked to carriers both achieve high immunogenicity and efficacy. However, end-linked glycoconjugates that contain long untethered stretches of native glycan chains may induce hyporesponsiveness by T cell-independent activation of B cells, while cross-linked medium-sized oligosaccharides may suffer from suboptimal saccharide epitope accessibility.

## Introduction

Bacterial polysaccharide (PS) molecules are versatile compounds that include lipopolysaccharides with their O antigens, capsular PSs, and exopolysaccharides. They play an important role as virulence factors of bacterial pathogens^1^ and protect the bacterium against host immune responses in at least three ways. First, surface PSs shield bacterial structures from the destructive activity of the alternative complement pathway of the innate immune system^2,3^. Second, capsular PSs also obstruct the classical complement pathway since antibodies cannot easily reach subcapsular bacterial surface proteins, thwarting subsequent complement deposition. Third, adaptive immunity to the bacterial PSs remains inefficient since saccharide structures are poorly bound by the antigen-presenting cells’ major histocompatibility complex (MHC), which leads to minimal T-helper cell-dependent antibody responses^4^. The latter probably explains why many host-dependent bacteria have developed surface carbohydrate structures during their evolution.

**Fig. 1:**
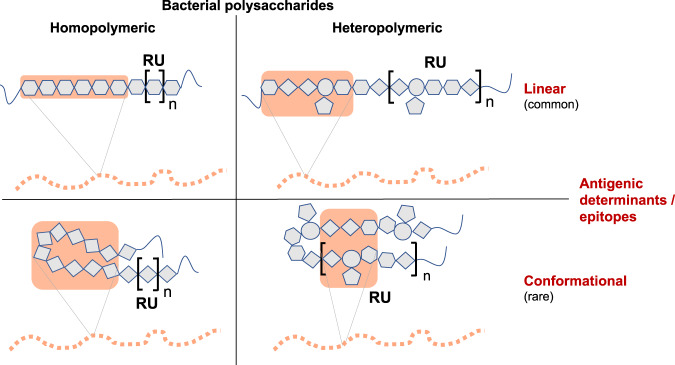
Key types of antigenic determinants (epitopes) of bacterial polysaccharides. In homopolymeric polysaccharides, identical monosaccharide residues repeat as a polymer. In heteropolymeric (or complex) PS, the repeat unit (RU, in square brackets) consists of diverse monosaccharide residues and may include branching side chains. The minimal required unit for antigenicity in linear epitopes consists of about 6–7 contiguous monosaccharides^121^. Linear epitopes may include the terminal monosaccharide residues of glycan chains. Conformational epitopes are formed by residues that are in close spatial proximity but dispersed across their primary sequence^122^. These require a sequence of residues sufficiently long to build or mimic the spatial conformations necessary for interaction with the antibody. As an example, the homopolymeric conformational epitope of the *Neisseria meningitidis* group B capsular PS antigen contains ten residues, although only the inner six residues interact with its cognate antibody^123,124^. Glycan fragments that exceed the length of the identified antigenic epitope are usually used as immunogens in glycoconjugate vaccines^125^.

Bacterial PSs also contribute to the ability of the microorganism to colonize specific ecological niches, resist antibiotics, and fight bacteriophages^5^. Their omnipresence in many bacterial pathogens has long generated interest in these compounds for vaccine development. The glycan chains of bacterial PSs form epitopes or antigenic determinants that can be recognized by B cell receptors (BCRs). These epitopes can be linear or conformational in nature and may consist of a minimum span of identical monosaccharides (in homopolymeric PS chains) or of various different monosaccharides including side chains (in heteropolymeric PS chains, Fig. 1). By the 1970s and 1980s, bacterial capsular PS vaccines became part of routine vaccinations, and licensed vaccines for adults based on purified PSs had been established for disease caused by *Neisseria meningitidis*, *Haemophilus influenzae* type b (Hib), *Salmonella enterica* serovar Typhi, and *Streptococcus pneumoniae*, among others^6,7^. Immunogenicity of those plain (i.e., unconjugated) PS vaccines is dependent on the capacity of long-chain PSs to cross-link surface immunoglobulin receptors on B cells^8–10^, leading to an antibody response with typical T cell-independent characteristics.

By themselves, bacterial PS generally induce only a relatively short-lived T cell-independent immune response and largely fail to activate maturation of memory B cells^11^ and long-lived plasma cells^12^. Consequently, they primarily induce low-affinity antibody responses and do not efficiently elicit long-term boostable immunological memory^9,13,14^. Because of their T cell-independent nature, plain PS vaccines such as those against Hib do not induce antibody responses and are therefore not effective in infants under 2 years of age^15–17^, with very few exceptions (such as the meningococcal serogroup A PS^18^). Fortuitously, conjugation of glycans to a carrier protein, a principle first investigated almost 100 years ago with pneumococcal PSs^19^, can convert these compounds to T cell-dependent antigens^20^ that induce high-affinity antibodies, antibody isotype switching, and a long-lasting memory immune response, making them efficacious in infants^21,22^. Coupling of a glycan to a carrier protein allows binding of the conjugate to saccharide-specific B cells, internalization of the conjugate, and presentation of carrier protein-derived peptide epitopes by MHC to T-helper cells. This eventually leads to clonal activation of the cognate B cells and their differentiation into plasma cells producing antibodies, with isotype switching to IgG and affinity maturation, and into memory B cells^21,22^.

All currently licensed glycoconjugate vaccines contain one of five carrier proteins. These are tetanus toxoid (TT), diphtheria toxoid (DT), a genetically modified cross-reacting material of the diphtheria toxin termed CRM_197_, meningococcal outer membrane protein complex OMPC, or *H. influenzae* protein D HiD^23^. However, a variety of alternative carrier proteins are also actively explored, such as modified versions of exotoxin A from *Pseudomonas aeruginosa*^24,25^, and the *S. pneumoniae* ABC transporter protein PiuA^26^.

**Fig. 2:**
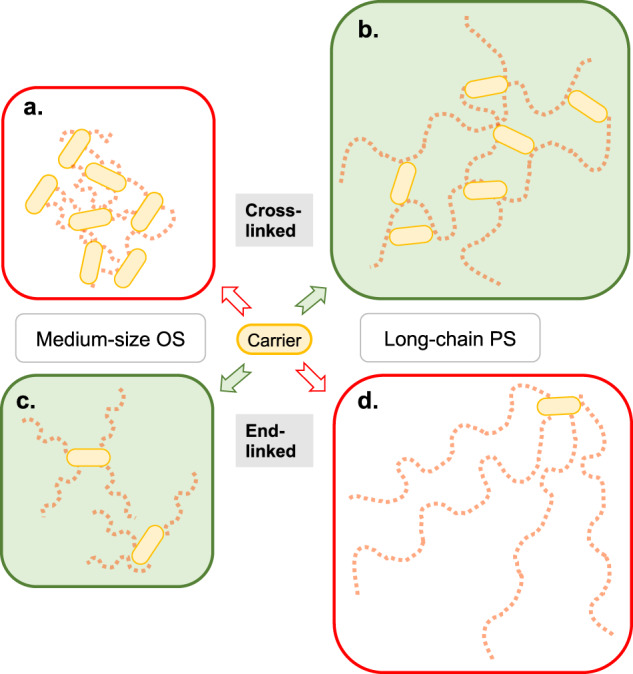
The four possible glycan activation categories. The basic structures of resultant glycoconjugates are shown. Glycan chains are depicted as strings of red squares, where each square indicates an epitope (see Fig. 1). Random activation of glycans results in cross-linked medium-sized oligosaccharide (OS) glycoconjugates (**a**) or cross-linked long-chain polysaccharide (PS) conjugates (**b**). Activation of glycan termini yields end-linked medium-sized OS glycoconjugates (**c**) or end-linked long-chain PS conjugates (**d**). Rarely performed activation of both ends of glycan chains may yield vaccines with a low-to-intermediate level of cross-linking. In current practice, **b** and **c** result in better-optimized glycoconjugate vaccines (see text for details).

Among the five carriers used in licensed vaccines, CRM_197_ and TT are by far the most used carriers. DT is often less potent,^27^ probably due to epitope inactivation during the chemical detoxification process, and both OMPC and HiD are rarely employed. The carriers are covalently bound to glycan molecules, either in end-linked configurations after activation of sugar moieties at glycan ends or as cross-linked substances following random activation of PS chains (Fig. 2).

Specific physicochemical properties of the glycoconjugate affect vaccine efficacy, and key parameters, such as the molecular size of the PS used for conjugation and the extent of the cross-linking, can have a profound impact on vaccine immunogenicity^28^. The number of attachment sites per carrier protein, e.g., needs to be sufficiently high to ensure efficient conjugation, limiting the level of residual free PS molecules. However, the degree of derivatization of carrier proteins requires careful optimization to avoid unwanted destruction of critical MHC epitopes within the protein sequence, which would abolish its T cell-stimulating activity. Selection of these key parameters as well as the choice of carrier protein and the activation and conjugation chemistries are influenced by the practicalities of the purification processes during vaccine production, where viscosity and molecular mass ratios of bound to unbound glycan may impact purity and yield of the final product^6^. As a consequence, different process chemistries and conjugation methods have been employed to prepare those conjugates. These chemistries include activation procedures to guide protein–glycan linkage sites and their numbers per molecule, as well as the introduction of spacer molecules between the two glycoconjugate components.

One of the primary quality attributes of microbial PSs obtained in vaccine production is the number of glycan repeating units per PS chain termed the degree of polymerization or chain length. This chain length is heavily affected by the production process. Often, the PSs are subjected to a size reduction from very large molecules (>500 kDa) to a heterogeneous mix of PSs of about 100–300 kDa prior to activation and chemical linkage to the specific carrier protein^29,30^. Size reduction of PS molecules improves subsequent conjugation efficiency in vaccine manufacturing.

The level of immunogenicity of plain bacterial PSs is correlated to their size and, therefore, their chain length^31,32^. Consequently, large PS size determination became a critical quality measurement to guarantee the immunogenicity of plain PS vaccines, probably explained by the need to cross-link BCRs to elicit T cell-independent antibody responses. But is this association also true for glycoconjugate vaccines? Unlike plain PS vaccines, induction of antibody mechanisms by glycoconjugates does not depend on cross-linking of BCRs. Instead, glycoconjugates are internalized by antigen-presenting cells, leading to T-helper cell responses triggered by carrier protein-derived peptides. Does a dependency on glycan chain length persist despite this difference? The following survey of the existing body of literature regarding the effect of chain length disparities on glycoconjugate vaccine efficacy was undertaken to clarify this question.

## Factors determining the glycan chain length of vaccine preparations

The size of the glycan chain in vaccine preparations is one important aspect of its structure. It is determined in various ways, utilizing terminology outlined in Table 1.

**Table 1:** Different parameters that characterize the glycan chain length of glycoconjugate vaccines.

Parameter	Abbreviation	Explanation	Select determining technologies
Repeating unit	RU	Number, identity, and linkage of the monosaccharides forming the repeating unit of a bacterial PS	Colorimetric assays
			Hydrolysis/derivation followed by chromatography such as high-performance anion-exchange chromatography with pulsed amperometric (HPAEC-PAD) or conductivity (HPAEC-CD) detection or gas chromatography with mass spectrometric detection (GC-MS)
			Nuclear magnetic resonance (NMR) spectroscopy (in various forms, such as 1D, 2D, proton, quantitative, and in combination with high-resolution magic-angle samples spinning probes)
Number of repeating units = degree of polymerization	#RU; DP	Number of repeating units in an OS or PS	*M*_w_ (glycan)/*M*_w_ (RU) 1 OS: obtained by measuring the ratio of total sugars over reducing end sugars (by colorimetric assay, GC-MS, MS/MS, or NMR) 2 PS: determined by measuring the ratio of Mw (glycan), obtained by high-performance size-exclusion chromatography with refractive index and multi-angle laser light scattering (HPSEC-MALLS/RI), over *M*_w_ (RU), calculated from its structure
Molecular weight	*M*/*M*_w_/*M*_r_	Molecular weight of the glycan, expressed in Daltons (Da) or gram per mole (g/mol)	Gel filtration and colorimetric analysis of fractions
			HPSEC-MALLS/RI

For further explanations of techniques and related references, see Hennessey et al. ^6^.

Two major aspects govern the length of the glycan chain in glycoprotein vaccine preparations—the underlying microbial genetics of the bacterial strain used to produce the glycan and the vaccine production process. These aspects are briefly discussed below.

### Microbial genetics

Depending on their genetic repertoire, bacterial strains can produce PSs of different chain lengths. To date, several of the bacterial regulators that govern the size of microbial glycans are known. Importantly, these can be genetically altered and swapped across species boundaries to generate glycans of altered lengths^33–35^.

Bacterial capsular PSs are, by and large, assembled either by Wzy-dependent polymerization or via the ABC transporter pathway. In Wzy-dependent polymerization, the PS is assembled in a stepwise manner in the bacterial periplasm from individual repeating units that are attached to a specific lipid carrier, undecaprenol diphosphate^36–39^. The so-called PS copolymerase proteins regulate the length of the polymer^40^. The best-studied of these enzymes, Wzz, has been known for a decade to affect the chain length of the O-antigen polymer in *Escherichia coli* lipopolysaccharide (LPS)^41^. In the ABC transporter pathway, however, the full-length PS is manufactured in the cytoplasm on a reducing terminal phosphatidylglycerol lipid bound to an oligosaccharide (OS) of β-linked KDO residues^36,39,42^. The chain length of PSs manufactured by this pathway is thought to be regulated in a geometry model by two interacting proteins, an extension enzyme called WbdA and a termination enzyme named WbdD. Overexpression of WbdD or diminished WbdA expression reduces chain length^43,44^. However, variations of this system have also been identified. As an example, polymerization, termination, and chain length regulation are performed by domains of a single protein, WbbB, in *Klebsiella pneumoniae* O12^45^. Similarly, *S. pneumoniae* serotype 3 uses a single membrane-bound synthase to produce its long-chain capsular PS. During polymerization, the nascent glycan remains tightly associated with the synthase until its release^46^, and enzyme catalytic phases may be affected by temperature and concentrations of the saccharide units^47^.

### The vaccine production process

#### Standard glycoconjugate vaccine production

Traditionally, glycoconjugate vaccines are produced by extraction of PSs from bacterial fermentation, followed by a multitude of purification and fragmentation steps, chemical activation, and subsequent conjugation to the carrier protein^48^. The carrier protein itself is similarly harvested from bacteria and subsequently purified. After chemical activation, it can be conjugated to the glycan via its naturally existing functional groups or on chemically introduced linker molecules.

**Fig. 3:**
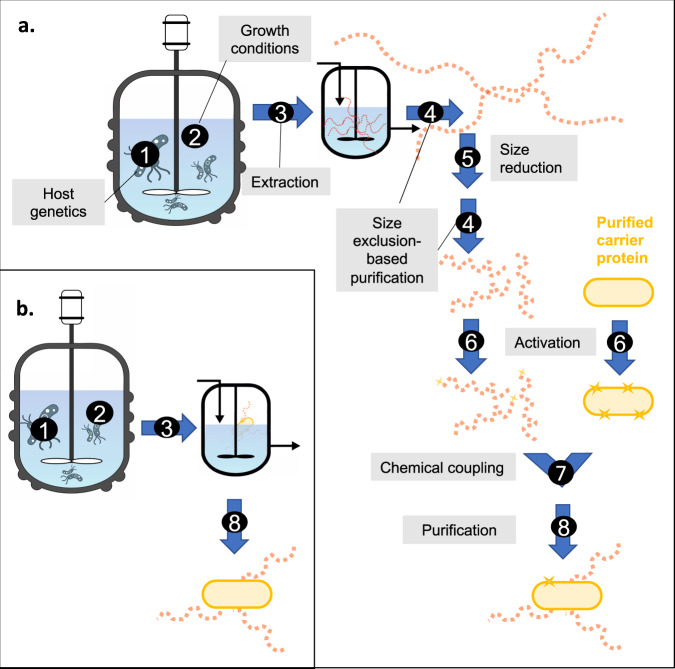
Glycan chain length depends on a number of factors and conditions applied during glycoconjugate vaccine production. **a** Standard glycoconjugate vaccine production process. **b** Bioconjugation process. Critical junctures are indicated by numbers. 1. Genetics of the bacterial strain employed. The bioconjugation process includes all genetic modifications introduced to the glycoconjugate production strain to facilitate in vivo coupling of the glycan to a carrier protein. 2. Growth conditions. 3. Strategies for isolation and purification of the glycan (or, in bioconjugation, of the conjugate). 4. Size-exclusion-based glycan or glycoconjugate purification procedures, such as tangential filtration (TFF) or size-exclusion chromatography. 5. Chemical or physical glycan size-reduction protocols, such as acid hydrolysis, ozonolysis, microfluidization, and/or periodate oxidation. 6. Activation chemistries of glycan and carrier protein. 7. Conditions applied for the chemical coupling of carrier and glycan. 8. Purification via combinations of chromatographic techniques, such as ion exchange and hydrophobic interaction.

There are a number of critical junctures in a production process that may affect the final size of the glycan in a vaccine formulation (Fig. 3). The chain length in a glycoconjugate vaccine manufactured by the traditional production process is determined not only by the genetic makeup of the microorganism but also by the fermentation conditions employed, including the carbohydrate source and the carbon/nitrogen ratio in the growth medium, the pH, the temperature, oxygen tension, and the levels of amino acids, vitamins, phosphate, and minerals^49^. Moreover, glycan isolation and purification conditions have a profound impact on the glycan chain length. Consequently, the molecular weight of meningococcal and pneumococcal capsular PSs, e.g., after industrial fermentation can range from approximately 100 to roughly 1000 kDa.

Often, the harvested PSs is subsequently chemically or mechanically fragmented^50^ and size-fractionated to alleviate glycan heterogeneity in the vaccine product^51,52^, to improve yields and consistency during purification processes, and to optimize the conjugation process. PSs are typically fragmented to about 100–300 kDa in cross-linking chemistries (usually still referred to as PSs) and to 5–20 kDa (typically termed OSs) in end-linking chemistry approaches^6^. The polysaccharide or OS is then chemically activated prior to conjugation, either randomly at hydroxyl groups or localized at the glycan end(s). Cross-linking or end-linking conjugation methods are subsequently used to achieve either random linkage along the glycan chain, or a selective attachment at the terminal end(s) of the sugar moiety, with or without spacer molecules^53^ (Fig. 2).

#### Bioconjugation

In an alternative technology called in vivo production of glycoproteins (aka protein glycan-coupling technology, or bioconjugation), the glycoconjugate is produced in a single step inside a microbe, where the glycan and the carrier protein are expressed and coupled inside the bacterial cell^54,55^, most often *E. coli*. Bioconjugation omits several necessary chemical reaction and purification steps that may hamper the yield, purity, and homogeneity of vaccine preparations produced by the traditional process. However, bioconjugation requires deliberate and careful genetic manipulation (bioengineering) of the host bacterium to introduce production of the glycan of choice (as a lipid-linked OS), the selected carrier protein, and protein glycosylation capability, often via dedicated plasmids^56^.

Bioconjugates are basically OS-based end-linked chemical glycoconjugates. Bacterial protein glycosylation is either oligosaccharyltransferase (OTase)-dependent (which can be N-linked, where glycans get attached to the amide nitrogen of asparagine residues, or O-linked, where glycans bond with the hydroxyl oxygen of serine or threonine residues) or OTase-independent^56^. In the former, glycans are moved en bloc from preassembled lipid-bound precursors to proteins in the periplasm. In the latter, glycosyltransferases move monosaccharides from nucleotide-activated precursors to sequentially form glycoproteins in the cytoplasm^56,57^. OTase-dependent strategies are (currently) much more prevalently applied, often using the N-linked OTase PglB from *Campylobacter jejuni* with a remarkable capacity to transfer a number of different PSs. However, several O-linked OTases are also actively investigated for use in bioconjugation processes, including pilin-specific OTases PglL, and PglS, the latter of which can transfer glycans with glucose at the reducing end^56,58^. In modern bioconjugation techniques, the carrier protein is also often genetically modified to include glycosylation sites in specific sequence patterns.

Sophisticated strategies have been developed that can control the length of the glycan chain of the glycoconjugate produced in these bacterial strains. Genetic manipulation of the chain length controller Wzz is an established strategy to increase the chain length of PS residues. Its overexpression can increase the production of a defined, long-sized PS population and decrease the production of short and very long saccharides^33^.

#### Other technologies

The glycan of a conjugate vaccine can also be synthetically (chemically) produced, such as in the anti-Hib vaccine *Quimi-Hib*^59,60^, where the repeat unit (RU) octamer of ribosylribitol phosphate (RRP)^61^ is end-linked to the carrier protein, TT. Unfortunately, the cost-effective manufacture of the necessary quantities of more complex synthetic OSs for standard vaccine production has remained a challenge. Enzymatic production of glycans is another alternative^52,62^, but has not permeated vaccine production pipelines, to date. These technologies can also be merged, as reported for the production of immunogenic meningococcal group C polysialic acid–TT glycoconjugates^63^. Other interesting alternative technologies currently considered for glycan vaccine purposes use outer membrane vesicles from engineered bacteria, the so-called generalized modules for membrane antigens (GMMAs), or high-affinity noncovalently linked avidin–biotin PS/protein complexes, creating macromolecular multiple antigen-presenting systems^48,64,65^. Furthermore, liposomes and gold nanoparticles are currently investigated as novel delivery vehicles for glycoconjugates, with encouraging results^52^.

### The impact of glycan chain length on vaccine immunogenicity

#### The minimal OS size to support immunogenicity

Efforts to determine the minimal glycan length to elicit immune responses are not only important from a cost perspective but are also useful in future research efforts to standardize and simplify vaccine production processes. OSs as short as tetramers have been shown to elicit effective immune responses, as components of an experimental *N. meningitidis* serogroup C (MenC) glycoconjugate^66^, as fragments of *S. pneumoniae* serotype 14 capsular PS conjugated to CRM_197_^67^, and as synthetic tetrameric RRP units conjugated to either TT or CRM_197_ for Hib^59^, the latter of which induced strong immune responses in nonhuman primates. For *N. meningitidis* serogroup A (MenA), randomly *O*-acetylated octamers were found to be sufficient to elicit protective antibodies in BALB/c mice^68^.

In tests using OSs representing the *Shigella flexneri* serotype 2a O antigen (OAg), the highest anti-LPS 2a IgG titer was generated by a glycoconjugate linked to TT that contained a chemically synthesized pentadecasaccharide that consisted of three RUs^69^. Subsequent studies showed that a minimum segment of nine sugar units is required to present the complete epitope to one of the protective antibodies^70^. Similarly, decasaccharide glycoconjugates elicited higher titers of anti-LPS IgG against the O-specific monomeric PS of *Vibrio cholerae* O1 serotype Ogawa than octamers or hexamers^71^. Consequently, the minimal oligo size capable of eliciting protective immune responses may depend, in part, on the RUs of the target PS and the steric characteristics that govern the size and structure of epitopes recognized by protective antibodies. A thorough understanding of these steric characteristics and their role in the interaction of the antigen with the host’s antibodies may help in future rational designs of synthetic glycans as components of novel glycoconjugate vaccines^7^.

Importantly, the minimal antigenic glycan epitope must be freely accessible to dock to the BCR’s immunoglobulin-binding cavity. In glycoconjugate vaccines, this can be achieved by the introduction of a spacer molecule between the antigenic epitope (glycan) and the carrier protein. These spacers enhance the exposure of antigenic epitopes on the surface of glycoconjugate vaccines and alleviate steric hindrances. They are (usually) small molecules with reactive groups on either end, which can be identical (such as in adipic acid, cystamine, or dithiobis(succinimidyl propionate)) or different (e.g., *N*-hydroxysuccinimidyl-3-maleinimidopropionate)^72^. However, many successful conjugates (both cross-linked long-chain PS and medium-sized end-linked OS formulations) do not employ spacers.

In addition, the immunogenicity profile of glycan epitopes that are composed of mostly homopolymers of monosaccharides with structural similarity to mammalian glycans is likely to differ from that of more complex glycan epitopes that contain unique pathogen-specific monosaccharides, many of which exist^73^. Therefore, the minimal glycan chain length to elicit a robust antibody response may depend on the exact serological characteristics of the PS.

#### The complex relationship between glycan length and immunogenicity

While glycan length undoubtedly impacts the immunogenicity of unconjugated PS vaccines, its impact on the performance of glycoconjugate vaccines seemed less clear, although early studies supported this viewpoint^74,75^. The complex relationship between these two parameters in glycoconjugates may be brought into better focus when separating end-linked constructs from cross-linked formulations.

#### End-linked constructs

An early clinical trial in the 1980s investigated the immunogenicity of two end-linked glycoconjugates against Hib, where one contained glycans of an average chain length of 8 RU and the other an average chain length of 20 RU^15^. While adults did not produce different titers of specific antibodies in response to vaccination with these different formulations, infants produced much more anti-polyribosylribitol phosphate (PRP) antibodies when the 20 RU formulation was used. Importantly, both of these conjugates were made without spacer molecules, which may have affected the accessibility of the glycan epitope for binding. Perhaps affecting interpretability even further, these constructs were end-linked on *both* ends of most glycan chains, presumably leading to a low-to-intermediate level of cross-linking in the final product.

The ostensible linear dependency of immunogenicity on glycan chain length was not corroborated in subsequent investigations on other end-linked formulations. Studies using glycoconjugate constructs of MenC PS fractions of different molecular weights (2-4, 4–10, and 10–50 kDa) conjugated to the carrier protein P64k did not identify differences in the immune response in BALB/c mice^76^. Moreover, a study using group B streptococcal (GBS) PSs conjugated with various end-linking chemistries to TT determined that immunogenicity of the preparations, as measured by IgG and opsonophagocytic titers in mice, increased with *decreasing* glycan size for GBS type II conjugates, whereas immunogenicity remained unaffected by glycan size for type III conjugates^77^.

A systematic investigation of the effect of chain lengths on the immunogenicity of primarily end-linked OS fragments of *S. pneumoniae* types 3, 6A, 18C, 19F, and 23F found little variation in antibody and opsonophagocytic titers generated by different conjugates that ranged in glycan molecular weights from 3 to 100 kDa^78^. However, a notable statistically relevant correlation was found for type 19F, where the shortest glycan fragments (about 8 kDa) generated by far the highest antibody titers in immunized rabbits, whereas native-sized conjugated glycan chains (100 kDa) displayed very poor immunogenicity, comparable with unconjugated native PS. The same study also confirmed that cross-linked native-length glycans (100 kDa) resulted in similar immunogenicity and opsonophagocytic killing as the end-linked construct produced with the shortest OS of *S. pneumoniae* type 6A (8 kDa).

Similarly, a study using end-linked conjugates of various sizes (10, 50, and 100 kDa) of capsular Hib PS to TT found that the shortest-length glycan achieved the highest immunogenicity of these preparations (as measured by IgG titers in rats), although statistical significance was not always achieved^79^. Finally, when testing several end-linked preparations of *S. enterica* serovar Typhimurium O-antigen (OAg)-CRM_197_ conjugates, those that were made with OAg of high molecular weight were considerably less immunogenic in mice than their counterparts of lower molecular weight, or those where the different HPLC-separated OAg preparations were mixed^80^.

A possible explanation for these observed phenomena was identified in a study that investigated end-linked glycoconjugate preparations of the *S. enterica* sv Typhi Vi antigen, coupled to CRM_197_, in mice. These constructs ranged in average glycan size from 9.5 kDa (about 37 RU) to 165 kDa (about 636 RU). The authors found that the conjugates with the longest glycan chains elicited a T cell-independent response (likely by cross-linking BCRs and, surprisingly, induced late apoptosis of Vi-specific B cells in spleen and early depletion of Vi-specific B cells in the bone marrow^81^. These detrimental effects were not observed in shorter-chain end-linked constructs, which induced a more prolonged proliferation of Vi-specific B cells in the spleen compared to their long-chain counterparts. The latter also generated a faster decline of Vi-specific IgG antibodies in mice than their smaller equivalents^81^.

Overall, these studies strongly suggest that medium-sized OS constructs result in better immunogenicity of end-linked glycoconjugates than long-chain PS formulations.

#### Cross-linked constructs

What about the effects of different glycan sizes on the performance of formulations that had been obtained using cross-linking chemistry? For group B type III streptococci, cross-linked glycoconjugate vaccine preparations of differing molecular glycan mass (approximate molecular weights of 349, 105, and 38 kDa) conjugated to TT produced different levels of specific IgG antibodies in mice. Titers largely correlated with saccharide length, although complete protection against a subsequent bacterial challenge was afforded by the two bigger saccharide fractions equally well^28^. A comparison of pentavalent pneumococcal cross-linked conjugate PS or OS vaccine preparations conjugated to CRM_197_ in infants showed that the PS formulations consistently produced higher anticapsular antibody titers, although not always in a statistically significant manner^82^. When comparing antibody titers generated by repeated vaccination of mice with pneumococcal type 4 PS- and OS-based cross-linked TT conjugates, the PS formulation resulted in significantly higher IgM and IgG titers compared with their OS counterparts^83^. Infants aged between 18 and 30 months generated higher antibody titers against *S. pneumoniae* types 6A and 23F when immunized with cross-linked glycoconjugates of higher chain length (3–5 RU versus 10–20 RU versus native PS)^84^.

When investigating full-length or fragmented Vi antigen cross-linked glycoconjugates bound to different carrier proteins (CRM_197_, DT, and TT), full-size fragments elicited higher amounts of anti-Vi IgG antibodies in mice after the first immunization—however, this advantage became statistically insignificant after the second immunization^85^. A study on glycoconjugates of the *Francisella tularensis* OAg of different sizes coupled to TT showed that cross-linked vaccine preparations from a genetically modified bacterium resulting in higher molecular weight (around 220 kDa) elicited a more protective immune response than low and native molecular weight preparations (25 and 80 kDa, respectively). Remarkably, the higher efficacy of the 220 kDa preparation was not due to superior antibody titers, but likely because of the antibodies’ improved affinity strength^35^.

These results all suggest that, unlike in end-linked glycoconjugates, in vaccines obtained with cross-linking conjugation chemistry, glycans of higher molecular weight may elicit a higher antibody response and/or improved protection. In cross-linked glycoconjugates, long-chain PS molecules may ensure optimal epitope density, with long glycan stretches that activate PS-specific BCRs. A carefully designed cross-linked long-chain conjugate vaccine avoids overly long glycan stretches. Such overly long chains may promote T cell-independent activation of B cells and subsequent hyporesponsiveness; the inability to mount an immune response after booster vaccination of at least the same magnitude as the response induced after primary vaccination^86^.

The most important parameter of a successful vaccine preparation remains its efficacy, i.e., whether it is able to prevent disease. Therefore, the next two chapters will briefly focus on the chain lengths of licensed glycoconjugate vaccines and investigate whether their efficacy correlates with glycan size.

#### Licensed *H. influenzae* vaccine preparations

A capsular plain PS vaccine against Hib (b-CAPSA), introduced and licensed in the United States in 1985, lacked efficacy in infants younger than 18 months, the primary target population^17^. Subsequently, the first glycoconjugate vaccine against this bacterium was licensed in the United States in December 1987. The product *ProHIBit* consisted of PRP cross-linked to DT (PRP-D)^87^. Three other Hib conjugate vaccine constructs soon obtained licensure—PRP cross-linked to OMPC (PRP-OMP, *PedVaxHIB*), PRP cross-linked to TT (PRP-T, *OmniHIB, Hiberix*), and RRP OSs of about 20 RU linked to CRM_197_ after activation of both glycan ends (HbOC aka *HibTITER*). A second glycoconjugate of RRP OS molecules linked to CRM_197_ was later produced with alternative chemistries, *Vaxem-Hib*. Finally, a vaccine consisting of chemically synthesized 8 RU of RRP end-linked to TT was also licensed, *Quimi-Hib*^60^ (Table 2). Consequently, licensed monovalent glycoconjugate vaccines against *H. influenzae* include long-chain PSs, medium-sized OSs, and short-chain octamers.

**Table 2:** Licensed monovalent glycoconjugate vaccines against Hib^a^.

Name	Current producer	Scientific compound nomenclature	First licensure	Glycan sizing	Glycan size	Glycan activation	Linker	Protein activation	Conjugation	Protein carrier
End-linked conjugation										
HibTITER^b^	Nuron Biotech^c^	PRP-CRM_197_, HbOC	1988	Periodate oxidation^d^	OS^e^	Periodate oxidation^d^	n.a.	n.a.	Peductive amination	CRM_197_
Vaxem-Hib^f^	GSK	PRP-CRM_197_	1995	Acid hydrolysis	OS	Amine^g^	NHS diester	n.a.	Amidation	CRM_197_
Quimi-Hib	Center for Genetic Engineering & Biotechnology	PRP-T, PRP-TT, sHbOT	2004	n.a.	OS^h^	Maleimide	3-Maleimidopropionate^i^	Thiol	Thioether	TT
Cross-linked conjugation										
ProHIBiT^j^	Sanofi	PRP-D, PRP-DT	1987	Heat	PS^k^	CNBr^l^	ADH	Active ester^m^	Amidation	DT
PedvaxHIB	Merck & Co.	PRP-OMP, PRP-OMPC	1989	n.a.	PS^k^	Bromide	Bigeneric spacer	Thiol	Thioether	OMPC
OmniHIB^n^	GSK	PRP-T, PRP-TT	1993	Alkaline hydrolysis^o^	PS^p^	CNBr^l^	ADH	Active ester^m^	Amidation	TT
ActHIB	Sanofi									
BioHib	Bharat Biotech									
novoHib	Panacea Biotec									
Hiberix	GSK									
SII HibPRO	Serum Institute of India	PRP-T, PRP-TT	2007	Alkaline hydrolysis^q^	PS^q^	CNBr^l^	ADH	Active ester^m^	Amidation	TT
HiBE	Biological E., Ltd.									

*Hib* *H. influenzae* type b, *n.a.* not available.

^a^Excludes combination vaccines.

^b^Vaccine was discontinued in 2007, originally developed by Pfizer.

^c^Currently in reorganization proceedings.

^d^Simultaneous depolymerization and activation, which cleaves between two contiguous hydroxyl groups (on the ribitol moiety) and activates both ends. The resulting conjugate is primarily end-linked, but can also contain cross-linked molecules, albeit to a much lesser extent than conjugates made from randomly activated PS.

^e^Approximately 20 RU.

^f^Vaccine discontinued in 2017, originally developed by Novartis.

^g^Reductive amination with diamino linker, activating reducing end of the OSs.

^h^Approximately 8 RU.

^i^Synthetic OS contains a built-in amino linker, extended by activation with *N*-hydroxysuccinimidyl 3-maleimidopropionate.

^j^Vaccine discontinued in 2000.

^k^Medium size, according to ref. ^88^.

^l^Random activation of hydroxyl groups.

^m^Forms in situ by *N*-ethyl-*N*′-(3-dimethylaminopropyl) carbodiimide (EDC) during conjugation.

^n^Vaccine discontinued, originally developed by Pasteur Mérieux Vaccins, replaced by Hiberix.

^o^Partial depolymerization during activation in an alkaline buffer.

^p^Large size, according to ref. ^88^.

^q^Controlled depolymerization to ~200 kDa^126^ during activation in an alkaline buffer.

Initial studies of the four primary licensed Hib vaccine constructs (PRP-D, PRP-T, and PRP-OMP, and HbOC) showed that all constructs but PRP-D generated mean serum antibody levels against PRP above 1 µg/mL^88,89^. The failure of PRP-D was likely related to its weaker carrier protein DT^27^. Notably, in a study of almost 500 American children, there were no statistically significant differences in the geometric mean antibody concentration after three doses of PRP-T or HbOC, with 95 and 91% of infants producing >1.0 µg/mL of antibody^90^. In addition to antibody measurements, efficacy data, the ultimate parameter for vaccine success, had been determined for the four constructs. Post dose 3, PRP-D was found to reach 90–100% efficacy when tested in thousands of Finnish children^91,92^, but only about 35% in Alaskan native infants^93^. For the OS-based formulation HbOC, efficacies had been determined to be 100% in two large studies with thousands of children^91,94^, while a smaller case–control trial in the United States determined 94.4% efficacy of HbOC after dose 3. For PRP-OMP, the efficacy of about 93% after dose 2 had been determined in a large trial involving Navajo and Apache native children^95^.

Subsequent studies investigated serum bactericidal antibody (SBA) titers of newer vaccine formulations and found that the end-linked OS-CRM_197_ formulation *Vaxem-Hib* usually outperforms the cross-linked-TT PS-containing glycoconjugate *ActHIB*, although both generated high titers suggesting long-term seroprotection^96–98^. In addition, the reported antibody concentrations raised by *Quimi-Hib*, the synthetic 8-mer vaccine, matched that of *Vaxem-Hib* 12 months after the first immunization series^60^.

In conclusion, efficacious Hib glycoconjugate vaccines were obtained by both medium-sized OS-based end-linked constructs and cross-linked long-chain PS-based formulations. Moreover, even short-sized end-linked synthetic glycans can yield a successful Hib vaccine.

#### Licensed *N. meningitidis* vaccines with group C glycoconjugates

Worldwide, several glycoconjugate vaccine preparations that include glycans directed against MenC have been licensed (Table 3). These are generally of superior immunogenicity compared to their unconjugated counterparts, with longer-lasting effects (e.g., refs. ^99–101^), and are licensed for use at an earlier age. The different conjugate vaccines are built using various chemistries, using different carrier proteins, and are used either as monovalent vaccines or as part of a combination formula targeting several serotypes of the same or various bacterial pathogens.

**Table 3:** Select licensed vaccines containing MenC glycoconjugates.

Name	Current producer	Scientific compound nomenclature	First licensed	Glycan sizing	Glycan size	Glycan activation	Linker	Protein activation	Conjugation	Protein carrier
End-linked conjugation										
Meningitec^a^	Nuron Biotech^b^	MenC-CRM_197_	1999	Periodate oxidation^c^	OS^d^	Periodate oxidation^c^	n.a.	n.a.	Reductive amination	CRM_197_
NeisVac-C	Pfizer	MenC-TT	2000	Periodate oxidation^c^	OS^d,e^	Periodate oxidation^c^	n.a.	n.a.	Reductive amination	TT
Menjugate/Meninvact	GSK	MenC-CRM_197_	2000	Acid hydrolysis	OS^g^	Amine^h^	NHS diester	n.a.	Amidation	CRM_197_
Menveo^f^		MenACWY-CRM_197_	2010							
Menactra^f^	Sanofi	MCV-4; MenACWY-D	2005	Acid hydrolysis^i^	PS^j^	n.a.^k^	ADH^l^	Active ester^m^	Amidation^n^	DT
MenQuadFi^f,o^		MenACWY-TT	2020	Periodate oxidation^c^	OS^(e)^	Periodate oxidation^c^	n.a.	n.a.	Reductive amination	TT
Cross-linked conjugation										
Menitorix^f^	GSK	Hib-MenC-TT	2005	n.a.	PS	CDAP^q^	ADH	Active ester^m^	Amidation	TT
MenHibrix^f^		Hib-MenCY-TT	2012							
Nimenrix^f,p^	Pfizer	MenACWY-TT	2012							

*MenC* *N. meningitidis* group C, *n.a.* not available.

^a^Originally developed by Pfizer.

^b^Currently in reorganization proceedings.

^c^Simultaneous depolymerization and activation, which requires two contiguous non-acetylated hydroxyl groups, and activates both ends. The resulting conjugate is primarily end-linked, but can also contain cross-linked molecules, albeit to a much lesser extent than conjugates made from randomly activated PS.

^d^DP of OS around 20–47^102,103^.

^e^De-*O*-acetylated OS; perhaps optional if within parentheses.

^f^Multivalent vaccine.

^g^DP of OS around 16^127^; an average molecular mass of MenC conjugates may be ~85 kDa^29^.

^h^Reductive amination with diamino linker, single-end activation of OS reducing end.

^i^Hydrogen peroxide-induced oxidative lysis was also reported^109^.

^j^Small size, approximately 20 kDa (∼50 RU)^109^.

^k^Single-end activation of OS reducing end.

^l^Coupled to PS aldehyde groups by reductive amination.

^m^Forms in situ by *N*-ethyl-*N*′-(3-dimethylaminopropyl) carbodiimide (EDC) during conjugation.

^n^The use of EDC also results in moderate levels of cross-linking due to a secondary amidation reaction between the MenC RU’s COOH groups and the carrier’s NH_2_ groups^110^.

^o^According to patent application US-2019/0175718-A1,^128^ process details parallel the Meningitec chemistry, with possible de-O-acetylation of the glycan.

^p^Originally developed by GSK.

^q^Random activation of hydroxyl groups.

At the turn of the century, three licensed OS-based MenC glycoconjugate vaccines existed—*Menjugate* (MenC-CRM_197_, end-linked, 14–19 RU), *Meningitec* (MenC-CRM_197_, primarily end-linked with a moderate level of cross-linking, 20–47 RU)^102^, and *NeisVac-C* (MenC-TT, primarily end-linked with a moderate level of cross-linking, 20–47 RU)^103^. When directly comparing the performance of these three vaccine formulations, both MenC-CRM_197_ vaccines were inferior to *NeisVac-C* in many studies^104–107^, and a hierarchy of *NeisVac-C* > *Menjugate* > *Meningitec* was usually observed. However, instead of glycan chain length, these differences in performance are perhaps associated with the fact that *NeisVac-C* used de-*O*-acetylated MenC OSs and TT as a carrier protein. Nevertheless, even *Meningitec* performed relatively well in absolute terms—a study evaluating its immunogenicity in over 100 British infants found that 98% of all participants achieved protective rabbit SBA titers of ≥1:8 post dose 3, with all of them achieving IgG titers of ≥2 µg/mL^108^.

*Menactra*, a meningococcal tetravalent ACWY conjugate vaccine, contains MenC glycans of roughly 20 kDa^109^, approximately 50 RU, end-linked to DT^110^. In trials encompassing hundreds of US-based infants, *Menactra* generated protective human SBA titers of ≥1:8 against MenC 30 days post dose 2 for 98.9% of participants^111^. The tetravalent ACWY end-linked mid-sized OS-based vaccine *Menveo* achieved the same level of human SBA protective titers ≥1:8 (82%) after a single dose as *Menactra* in a phase III study involving over 1000 adolescents^112^. In addition, phase III trials of the most recent addition to the arsenal of end-linked multivalent size-reduced MenC glycoconjugate vaccines, *MenQuadFi*, confirmed its non-inferiority compared to *Menactra* and *Nimenrix* and suggested a superior seroresponse against MenC, with protective human SBA titers 30 days after immunization in >99% of naive or preimmunized toddlers 12–23 months of age^113^, and >88% in adolescents and adults^114^.

There is only one MenC glycoconjugate construct that uses native PS, skipping a targeted glycan sizing step entirely. This construct is used in several cross-linked polyvalent vaccine formulations (*MenHibrix, Nimenrix*, and *Menitorix*). Immunogenicity experiments comparing *MenHibrix* with the OS-based end-linked MenC-CRM_197_ monovalent vaccine (*Menjugate*) in hundreds of healthy infants suggested no significant difference in rabbit SBA titers after the third dose^115^. Similarly, *Nimenrix* showed favorable immunogenicity profiles after single and double injections, with 100% of more than 200 participating infants obtaining rabbit SBA levels ≥1:8 against MenC 1 month after the dose, and 90.8% showing that level 3 years post dose^116^.

In summary, end-linking technologies with medium-sized glycans as well as cross-linking chemistries with long-chain glycans lead to efficacious MenC conjugates in licensed vaccines with no obvious correlating differences in vaccine immunogenicity. This observation mirrors the data collected for Hib glycoconjugates, above.

## Conclusions

**Fig. 4:**
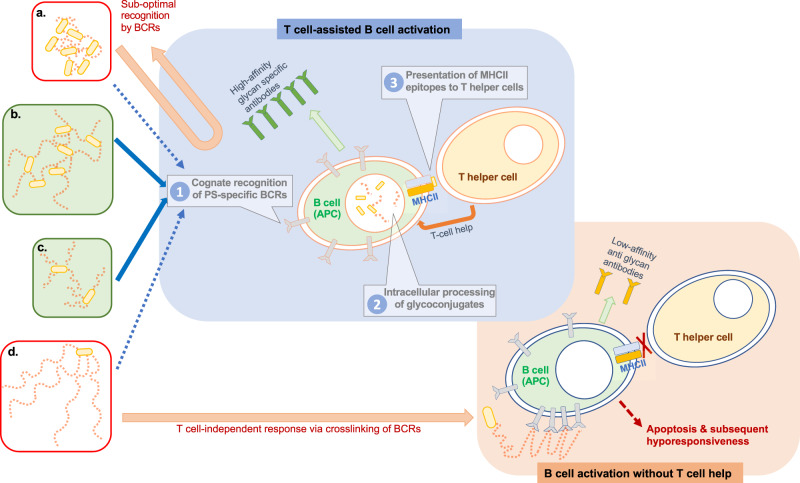
A hypothetical model of the role of chain length in the interaction of glycoconjugates with antigen-presenting B cells (APC) and T helper cells. The necessary steps for glycoconjugate vaccines to engender B cell maturation and production of glycan-specific antibodies are enumerated in the central blue segment. Glycoconjugate construct (**a**) contains medium-sized glycan chains that are excessively cross-linked and therefore not sufficiently accessible to be optimally recognized by the B cell receptors (BCRs). Instead, functional vaccines can be achieved by rational design of linkage sites and chain lengths in the vaccine molecule. Appropriate levels of cross-linking of long-chain polysaccharides (construct **b**) or end-linked chemistries for medium-sized oligosaccharides (construct **c**) can lead to protective glycoconjugate vaccines. In those settings, T-helper cells recognize the MHCII-bound peptide complexes and become activated. With T-helper cell support, cognate B cells mature to memory B cells and produce glycan-specific antibodies. However, excessively long glycan stretches (construct **d**) may act similar to unconjugated PS and cross-link BCRs, generating mainly T cell-independent immune responses resulting in hyporesponsiveness. Without T-helper cell support, B cells become temporarily activated but proceed to undergo apoptosis, impeding the production of long-lasting memory B cells and long-lived plasma cells^81^.

A thorough review of the literature suggested that cross-linked glycoconjugates may require long-chain PSs for optimal immunogenicity, whereas end-linked constructs may benefit from medium-sized OSs. Either strategy can achieve an optimal density of native saccharide epitopes, allowing strong immune responses. Short chains of too few saccharide molecules may not contain an adequate density of accessible epitopes, while saccharide chains that are too long and formulations containing an excess of unconjugated PS molecules may carry the risk of promoting T cell-independent activation of B cells^81^ by BCR cross-linking (Fig. 4). The model relies on the stimulation of either carrier-based peptide T-helper responses (the classical paradigm for conjugate vaccines) or carbohydrate-specific CD4^+^ T cell clones (Tcarbs) to produce the necessary cytokines for B cell activation^117^, a concept we eagerly await to be consolidated by future research.

Other factors determining vaccine efficacy may lie in the specific conformational features of both the carrier and the glycan components, and in specific features of the host, such as their metabolic state and the degree of senescence or maturity of their immune response system. Chain density per carrier protein molecule has also long been suspected to be of vital importance^15,78,118,119^ and is heavily influenced by the process chemistries applied during vaccine preparation. Peak glycoconjugate performance may require the anchoring of minimal stretches of contiguous intact glycan epitopes to carrier proteins in a ratio that optimizes epitope loading for each protein molecule. At the optimal ratio, the carrier/glycan hybrid molecules maximally activate T helper cells.

In our survey, the clinical data on licensed glycoprotein vaccines of various glycan chain lengths do not indicate a relevant difference in protection, the ultimate goal of vaccination. We conclude that saccharide stretches in glycoconjugate vaccines need to be sufficient but not overly long for optimal vaccine performance. This crystallized paradigm can be delivered either via end-linked OS conjugates or via cross-linked PSs. This implies that successful glycoconjugate vaccines do not require long-chain lengths—an important realization that supports the future development of vaccines based on synthetic OSs that are linked to carriers via spacer molecules. Other technologies, such as GMMA and affinity non-covalent coupling, may also flourish^65,120^. The search for optimal glycan chain lengths (or, more precisely, the optimal number of contiguous epitopes between carrier proteins of glycoconjugates) will likely continue and be driven by efforts to minimize the size of each molecule without a loss (and possibly with gain) of function. In short term, though, bioconjugation using medium-sized OSs and direct enzymatic end-linking may offer the best balance between efficiency, simplicity, and optimal immunogenicity of glycoconjugate vaccines.
